# Supplementary material for: Gestational Exposure to Bisphenol A Affects Testicular Morphology, Germ Cell Associations, and Functions of Spermatogonial Stem Cells in Male Offspring
Source: Int J Mol Sci. 2020 Nov 17;21(22):8644. doi: 10.3390/ijms21228644 (PMC7696188; doi:10.3390/ijms21228644)
Supplement: Supplementary file 1 [file ijms-21-08644-s001.pdf]

International Journal of Molecular Sciences

Article

Gestational Exposure to Bisphenol A Affects Testicular Morphology, Germ Cell Associations, and Functions of Spermatogonial Stem Cells in Male Offspring

Polash Chandra Karmakar [1,†], Jin Seop Ahn [1,†], Yong-Hee Kim [1], Sang-Eun Jung [1], Bang-Jin Kim [2], Hee-Seok Lee [3], and Buom-Yong Ryu [1,*]

[1] Department of Animal Science and Technology and BET Research Institute, Chung-Ang University, Anseong, 17546, Korea; polashmicro@gmail.com (P.C.K.); ahnjs@cau.ac.kr (J.S.A.); yhkcau@naver.com (Y.-H.K.); tkddms2428@naver.com (S.-E.J.)
[2] Department of Cancer Biology, Perelman School of Medicine, University of Pennsylvania, Philadelphia, PA, 19104, USA; bakim@pennmedicine.upenn.edu
[3] Department of Food Science & Technology, Chung-Ang University, Anseong, 17546, Korea; hslee0515@cau.ac.kr
* Correspondence: byryu@cau.ac.kr; Tel.: +82-31-670-4687; Fax: +82-31-670-0062
† These authors contributed equally to this work.

Supplementary Methods

Collection of testis and determination of testicular abnormalities

F1 male mice were selected from each dam and sacrificed at PNDs 30, 60, and 120, following which the testes were collected and weighed. Testes of F2 and F3 males were collected at PND 120. The testes were dissected vertically into two parts: one for paraffin sectioning and the other for fluorescence-activated cell sorting (FACS). Testis parts used for sectioning were fixed in Bouin's solution at room temperature for 6 h and subsequently washed with 70%–100% ethanol gradient to dehydrate the tissue at intervals of 5 min, followed by washing with xylene. Testis tissues were embedded in paraffin wax, and five micrometer-thick serial sections were cut and placed on glass slides. Some of the slides were stained with hematoxylin and eosin for examination of testicular morphology under a microscope (TE2000-U, Nikon, Chiyoda-ku, Tokyo, Japan). Seminiferous tubules (STs) with huge lumen or with no lumen, abnormal cell mass inside the lumen area, and germ cell loss in the seminiferous epithelium (SE) or presence of vacuoles in the SE were considered as testicular abnormalities. The measure of testicular abnormalities was examined according to a procedure previously described by Doyle et al., 2013 [1]. All STs in one section were considered for obtaining data.

Flow cytometric analysis

Testicular cell populations (classified as 1C-, 2C-, and 4C-DNA content subpopulations, based on their DNA content) were measured using FACS. Part of the mouse testes were used for testicular cell separation, according to Oatley and Brinster [2]. In brief, tunica albuginea was removed and the testis tissue containing STs was treated enzymatically with collagenase (1 mg/mL; Gibco, CA, USA) for 1-2 min at 37°C. STs were then treated with a solution containing a 4:1 ratio of 0.25% trypsin-EDTA (Gibco) and Dulbecco's phosphate-buffered saline (DPBS)-dissolved DNase I (7 mg/mL; Roche, Mannheim, Germany) at 37°C for 5-6 min with flick mixing. Fetal bovine serum (FBS) (10% [v/v], Thermo Fisher Scientific, Utah, USA) was used to inactivate the enzymatic action, following which a cloudy suspension was prepared by slow pipetting. The cell suspension was filtered using a nylon mesh of 40-μm pore size (BD Biosciences, San Jose, CA, USA). The cells were then fixed with 70% chilled ethanol and stored at 4°C overnight. The fixed cells were washed twice with chilled DPBS (Gibco). Finally, the cells were treated with 500 μg/mL RNase (Sigma-Aldrich, St. Louis, MO, USA) and 0.1% Triton X-100 (v/v, in DPBS), stained using propidium iodide (Sigma-Aldrich), and analyzed using a FACS Calibur Flow Cytometer (BD Biosciences) according to Liu *et al.* [3].

Germ cell transplantation for evaluating SSC activity after BPA exposure

The transplantation procedure was conducted according to a previously described protocol [2,4]. Most of the effects related to BPA exposure were observed in F1 offspring and at NOAEL and LOAEL doses. Therefore, F1 males exposed to these two doses were selected as germ cell donors for this

experiment along with control and positive control (EE-exposed) groups. Six-week-old CD-1 male mice were selected as recipients. Recipient mice were prepared 6 weeks before the day of transplantation by injecting busulfan into the intraperitoneal cavity at a concentration of 35 mg/kg body weight to eradicate endogenous germ cells and spermatogenesis from the recipient testes.

Collection of germ cells from donor mice was conducted just before transplantation. Donor mice were sacrificed, their testes were collected, and tunica albuginea from the testis was removed. Testicular germ cells were separated using the same procedure described in the 'Flow cytometric analysis' section. After filtration with a 40-μm pore sized mesh, the filtered cells were stained using a membrane linker dye (PKH26; red fluorescence, Sigma-Aldrich) at a concentration of 3.2×10^{-6} M and by following the manufacturer's protocol. The proportion of SSCs in a mouse testis is ~0.01% of the total testicular cells [5]. Therefore, we prepared a cell suspension at a concentration of 50×10^{6} cells/mL with a solution containing 10% (v/v) FBS and 10% DNase I (7 mg/mL) dissolved in minimum essential medium α (Gibco). Following that, the recipient mice were anesthetized using 75 mg/kg ketamine and 0.5 mg/kg medetomidine before transplantation. A hair trimmer was used to remove the lower abdominal hair and the area of surgery was disinfected using iodine and ethanol (70%). A small surgical wound was made, and the testis was driven out carefully from the abdomen. The suspension of donor germ cells was then labeled with 7% (v/v) trypan blue and injected into the recipient testes through efferent ducts, as described previously [6]. About 8-10 μL (~5.0×10^{5} cells) of donor germ cell suspension was injected into each testis, which filled ~80% of the surface seminiferous tubules.

One month after transplantation, the recipient mice were euthanized and the collected testes were visualized under a fluorescence microscope (AZ100, Nikon). The testes were then decapsulated and the tubules were dispersed gently. The donor cell-derived colonies were visualized separately and counted, as described previously [7]. Spherical PKH26 positive colonies, at least 200 μm or greater in length, were considered to have been produced from one SSC and counted. Some testes were recovered, cryosectioned, and histologically observed under a fluorescence microscope to confirm the proliferation of spermatogonia at the basement membrane of recipient seminiferous tubules and complete spermatogenesis and spermiogenesis.

Supplementary Figure

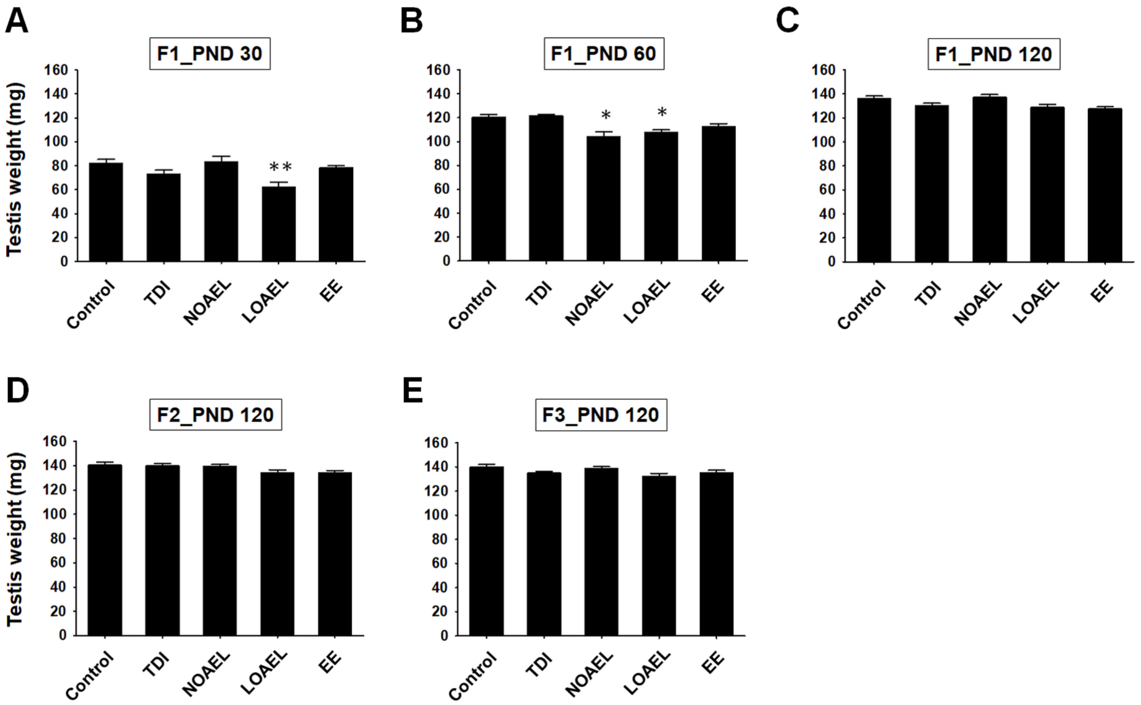

Figure S1. Weight of the testes of F1 offspring at postnatal day (PND)s (A) 30, (B) 60, and (C) 120 have been represented as a bar graph (n=20 mice/group). Testis weight of (D) F2 and (E) F3 offspring at PND 120 (n=20 mice/group). Values with superscript character (*) indicate significant differences (analyzed using one-way analysis of variance, ANOVA) compared to control ($*p<0.05$ and $**p<0.01$).

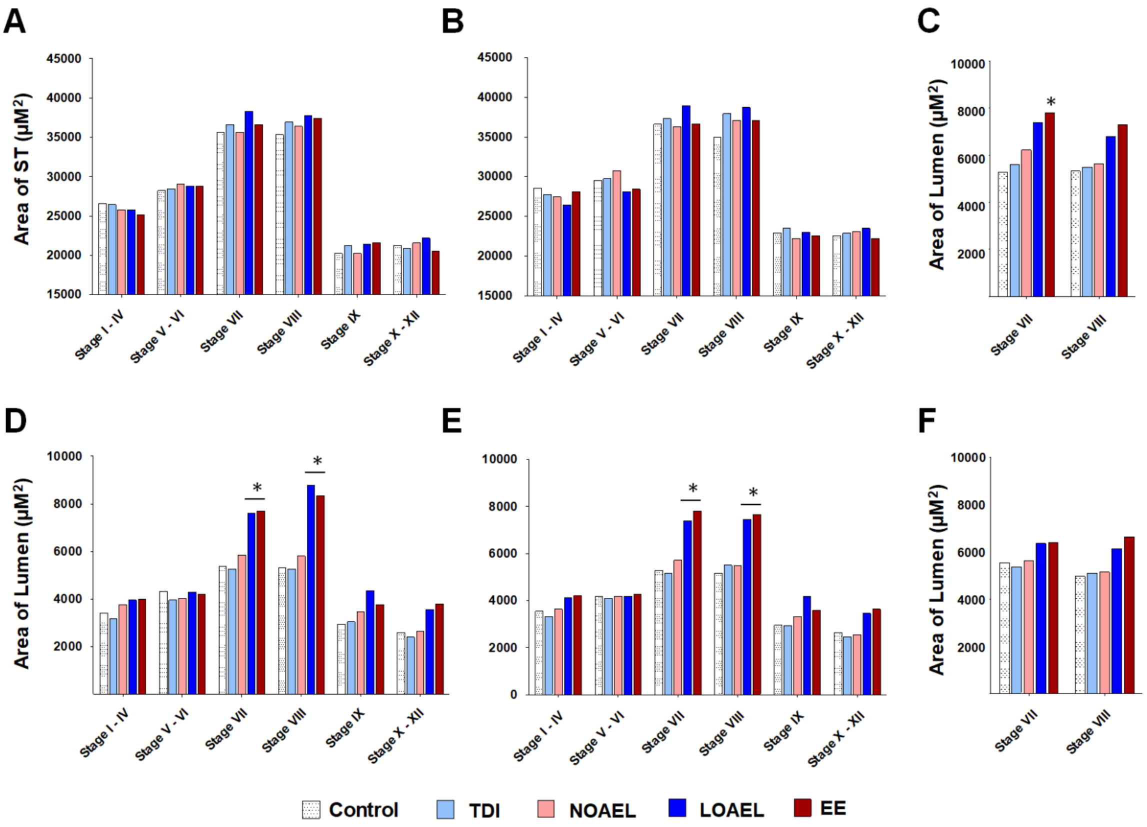

Figure S2. Areas of seminiferous tubules and lumen. As the area of seminiferous tubules (STs) and lumen differ in stages of seminiferous epithelium (SE), the above graphs are showing the ST and lumen areas at different SE stages. Areas of the STs in F1 offspring at postnatal day (PND)s (A) 60 and (B) 120 were measured in μm^2. The areas of lumen (μm^2) in F1 offspring were measured at PNDs (D) 60 and (E) 120. Around 15 mice/group were used to generate this data. Areas of lumen (μm^2) in (C) F2 and (F) F3 offspring at PND 120 (n=15 mice/group). Data have been analyzed using one-way analysis of variance (ANOVA) and asterisk (*) indicates significant differences between exposure and control groups at the same stage of SE (*$p<0.05$).

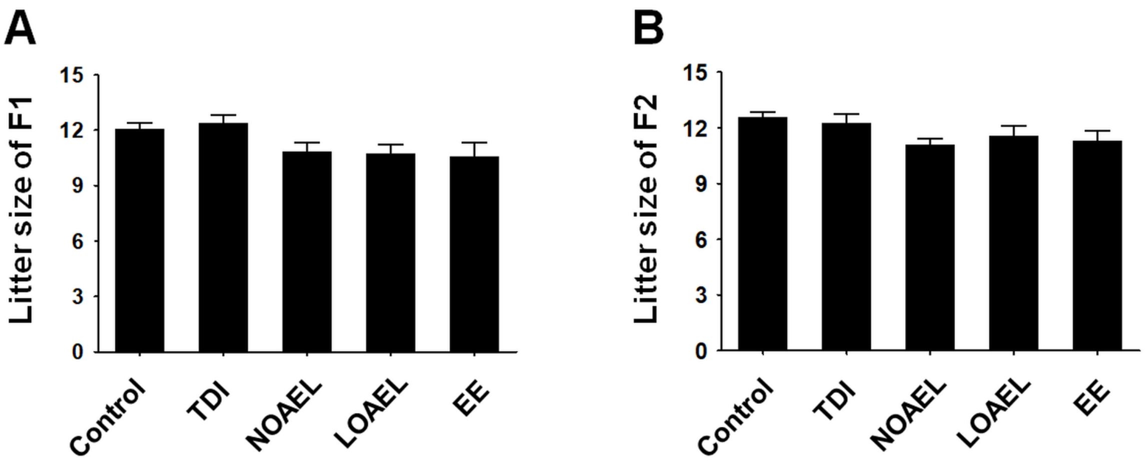

Figure S3. Litter sizes generated from F1 and F2 offspring. Total number of pups was calculated for litter size at their postnatal day (PND) 1. Bar graphs represent the litter size of (A) F1 and (B) F2 offspring.

Supplementary Table

Table S1. Anogenital distance (AGD), nipple retention and survival rates of F2 and F3 male offspring.

Generation	Treatment group	Male pups (n)	AGD	Nipple retention (%) [PND 10]*	Survival rates (%) [PND 21]
F2	Control	21	2.34 ± 0.54	4.81 ± 0.25	96.13 ± 1.21
	TDI	22	2.19 ± 1.04	4.70 ± 1.60	95.17 ± 0.71
	NOAEL	24	2.12 ± 1.23	5.05 ± 0.40	94.75 ± 1.01
	LOAEL	25	2.23 ± 0.35	5.11 ± 1.57	94.67 ± 1.16
	EE control	29	2.30 ± 0.99	5.88 ± 1.06	94.11 ± 0.55
F3	Control	15	2.29 ± 0.65	4.80 ± 0.88	95.24 ± 0.81
	TDI	17	2.28 ± 1.11	4.90 ± 1.11	94.05 ± 0.71
	NOAEL	17	2.25 ± 1.09	5.01 ± 1.40	94.75 ± 1.22
	LOAEL	18	2.19 ± 0.39	4.99 ± 1.21	93.62 ± 1.16
	EE control	20	2.28 ± 1.07	5.08 ± 1.17	94.02 ± 1.68

* Data generated from male pups only

Supplementary References

1. Doyle, T.J.; Bowman, J.L.; Windell, V.L.; McLean, D.J.; Kim, K.H. Transgenerational Effects of Di-(2-ethylhexyl) Phthalate on Testicular Germ Cell Associations and Spermatogonial Stem Cells in Mice. *Biol. Reprod.* **2013**, *88*, 1–15, doi:10.1095/biolreprod.112.106104.
2. Oatley, J.M.; Brinster, R.L. Spermatogonial stem cells. *methods Enzym.* **2006**, *419*, 259–282, doi:10.1016/S0076-6879(06)19011-4.
3. Liu, C.; Duan, W.; Li, R.; Xu, S.; Zhang, L.; Chen, C.; He, M.; Lu, Y.; Wu, H.; Pi, H.; et al. Exposure to bisphenol A disrupts meiotic progression during spermatogenesis in adult rats through estrogen-like activity. *Cell Death Dis.* **2013**, *4*, e676, doi:ARTN e676 10.1038/cddis.2013.203.
4. Brinster, R.L.; Zimmermann, J.W. Spermatogenesis Following Male Germ-Cell Transplantation. *Proc. Natl. Acad. Sci. U. S. A.* **1994**, *91*, 11298–11302, doi:DOI 10.1073/pnas.91.24.11298.
5. Nagano, M.C. Homing efficiency and proliferation kinetics of male germ line stem cells following transplantation in mice. *Biol. Reprod.* **2003**, *69*, 701–707, doi:10.1095/biolreprod.103.016352.
6. Ogawa, T.; Aréchaga, J.M.; Avarbock, M.R.; Brinster, R.L. Transplantation of testis germinal cells into mouse seminiferous tubules. *Int. J. Dev. Biol.* **1997**, *41*, 111–122, doi:10.1387/ijdb.9074943.
7. Nagano, M.; Avarbock, M.R.; Brinster, R.L. Pattern and Kinetics of Mouse Donor Spermatogonial Stem Cell Colonization in Recipient Testes. *Biol. Reprod.* **1999**, *60*, 1429–1436, doi:10.1095/biolreprod60.6.1429.
